# Interaction Effects of Light, Temperature and Nutrient Limitations (N, P and Si) on Growth, Stoichiometry and Photosynthetic Parameters of the Cold-Water Diatom *Chaetoceros wighamii*

**DOI:** 10.1371/journal.pone.0126308

**Published:** 2015-05-20

**Authors:** Kristian Spilling, Pasi Ylöstalo, Stefan Simis, Jukka Seppälä

**Affiliations:** 1 Finnish Environment Institute, Marine Research Centre, PO Box 140, Helsinki, Finland; 2 Tvärminne Zoological Station, University of Helsinki, J.A. Palménin tie 260, Hanko, Finland; 3 Plymouth Marine Laboratory, Plymouth, United Kingdom; Mount Allison University, CANADA

## Abstract

Light (20-450 μmol photons m^-2^ s^-1^), temperature (3-11°C) and inorganic nutrient composition (nutrient replete and N, P and Si limitation) were manipulated to study their combined influence on growth, stoichiometry (C:N:P:Chl *a*) and primary production of the cold water diatom *Chaetoceros wighamii*. During exponential growth, the maximum growth rate (~0.8 d^-1^) was observed at high temperture and light; at 3°C the growth rate was ~30% lower under similar light conditions. The interaction effect of light and temperature were clearly visible from growth and cellular stoichiometry. The average C:N:P molar ratio was 80:13:1 during exponential growth, but the range, due to different light acclimation, was widest at the lowest temperature, reaching very low C:P (~50) and N:P ratios (~8) at low light and temperature. The C:Chl *a* ratio had also a wider range at the lowest temperature during exponential growth, ranging 16-48 (weight ratio) at 3°C compared with 17-33 at 11°C. During exponential growth, there was no clear trend in the Chl *a* normalized, initial slope (α*) of the photosynthesis-irradiance (PE) curve, but the maximum photosynthetic production (P_m_) was highest for cultures acclimated to the highest light and temperature. During the stationary growth phase, the stoichiometric relationship depended on the limiting nutrient, but with generally increasing C:N:P ratio. The average photosynthetic quotient (PQ) during exponential growth was 1.26 but decreased to <1 under nutrient and light limitation, probably due to photorespiration. The results clearly demonstrate that there are interaction effects between light, temperature and nutrient limitation, and the data suggests greater variability of key parameters at low temperature. Understanding these dynamics will be important for improving models of aquatic primary production and biogeochemical cycles in a warming climate.

## Introduction

Models of phytoplankton growth are important for understanding aquatic production and ecosystem-scale biogeochemistry. Abiotic variables such as light, temperature and nutrient availability are the most important aspects regulating productivity and growth in phytoplankton. The influence of these parameters on production is most often studied independently, whereas interaction effects such as the temperature dependent nature of light utilization for photosynthesis, may be expected [1].

The Redfield C:N:P ratio of 106:16:1 is widely used as an average composition of these elements in phytoplankton [2]. However, different cellular components have specific stoichiometric fingerprints and growth rate will affect the ratio between different elements [3]. For example, there tend to be greater allocation of resources to P rich RNA during exponential growth (reducing the N:P ratio), and the N:P ratio has different optima for different growth conditions; the canonical N:P of 16 represents rather an average of a whole community than the optimum for individual species [4]. Nutrient limitation will typically move the stoichiometric ratio even further away from the Redfield ratio as the limiting nutrient is at a minimum and non-limiting nutrients are taken up and stored in excess [2].

The relationship between carbon and chlorophyll *a* (C:Chl *a* ratio) is central in modelling global carbon fluxes due to the Chl *a* retrieval capability from global ocean-color remote sensing. This ratio is highly dynamic, depending on environmental variables such as light and temperature [5], which should be taken into account when modeling ocean biochemical processes [6]. Light is the fundamental driver of carbon fixation in the ocean, and phytoplankton optimize primary production by regulating their photosynthetic pigments, i.e. photoacclimation. Most oceanic biogeochemical models include dynamic C:Chl *a* ratios with photoacclimation parameterization [1,7–8], and it is important to understand interaction effects of several environmental parameters for improved parameterization [9–10].

When modelling primary productivity, some of the key parameters in measurements of photosynthetic production are: the maximum light utilization coefficient (α*), which is the initial slope, α, of the photosynthesis-irradiance (PE) curve normalized to Chl *a*; the maximum photosynthetic rate (P_m_*); the irradiance where production equals consumption i.e. the compensation light intensity (E_c_); and finally the light saturation parameter (E_k_) [11].

The photosynethic quotient (PQ) is given as the molar ratio of oxygen (in the form of O_2_) produced per C fixed by photosynthesis. The PQ is normally >1, indicating that a fraction of the reducing power created in the light reaction is used for other purposes than C fixation in the Calvin-Benson cycle, e.g. lipid or protein synthesis [12]. Furthermore, when the N source is nitrate that needs to be reduced, the PQ will be higher compared to a situation where ammonium is the N source [13]. Another process that will affect the PQ is photorespiration, which is a process consuming the O_2_ produced during photosynthesis and thereby lowering the PQ [14].

We may expect climate change to disproportionally affect regions with strong seasonality in light availability and surface water temperature. Thus, it is important to improve biogeochemical models particularly in regions where seasonal primary production is coupled to low temperatures, such as seas and oceans at high latitudes. Most of the focus on interaction effects between different environmental variables stems from work on lakes [15–16]. Coastal areas in arctic or subarctic regions will be subjected to many of the same changes, but relatively few studies have addressed interaction effects in these areas, in particular for cold water adapted phytoplankton species [16–17].

In this study, we present growth, element stoichiometry and primary production of a cold-water adapted, model organism, *Chaetoceros wighamii*; a common bloom forming diatom in the Baltic Sea [18], subjected to a range of growth conditions around its known optimum. Our goal was to investigate potential interaction effects between light, temperature and nutrient limitation, and the results provide generically applicable productivity data for a cold-water diatom.

## Materials and Methods

### Culture acclimation and growth

*Chaetoceros wighamii* was adopted from the culture collection of the Tvärminne Zoological Station (strain TVCWI) and cultured in T2 medium at 6 PSU, which is a modified f/2 medium [19] with N:Si:P nutrient ratios adjusted to 16:8:1, previously suggested to be close to optimal for this diatom [18]. The batch culture was grown in 2L polycarbonate flasks (filled to 1.5 L) and acclimated to different temperature (3, 7, 11 and 15°C) and irradiance (20, 40, 130 and 450 μmol photons m^-2^ s^-1^) from daylight, fluorescent tubes (Philips TLD 965). Light was provided using a 16:8 hour light-dark cycle. The flasks were held in a temperature-regulated water bath and irradiance was adjusted with neutral density screens. The cultures were kept in suspension by bubbling with pre-filtered (0.2 μm) air. Growth was monitored daily by counting cells with a FlowCam (FluidImaging), which collects micrographs of individual cells passing through a flow cuvette. The growth rate was calculated from a linear fit to natural logarithm (ln) transformed cell numbers, and all the fits are presented in the supporting information (S1 Fig). The first set of measurements of particulate organic carbon (POC), nitrogen (PON) and phosphorus (POP), chlorophyll *a* (Chl *a*) and photosynthesis-irradiance (PE) curves were obtained during exponential growth. The biomass during the exponential growth sampling was approximately 1000 μmol POC L^-1^ and 500 μg Chl *a* L^-1^, which was approximately 10% of the maximum (in terms of POC) during the stationary growth phase.

After the sampling, all but 100 mL of the culture was removed and new medium set up to produce N, P or Si limitation was added. Nutrient limitation was ensured by increasing all but the limiting nutrient to 5-fold concentration. This procedure was carried out for a subsample of initial treatments, representing 5 different temperature and light conditions (Table 1). Growth was monitored as described above until cell abundance did not increase over minimum 3 consecutive days. A second set of measurements was taken during this early stationary growth phase. The growth curves until the point of harvesting are presented in the supporting information (S2, S3 and S4 Figs for N, P and Si limitation respectively). The average biomass during sampling of the stationary growth phase was 11200 μg POC and 3800 μg Chl *a*. Photosynthetic parameters were not measured under Si limitation due to time constraints.

**Table 1:** Growth rate, stoichiometry and photosynthetic parameters for *Chaetoceros wighamii* under different growing conditions.

Growth conditions			Growth rate	Stoichiometry					Photosynthetic parameters (O_2_)					
Temp	Light	Growth	(d^-1^ ± SE)	C:N	C:P	C:Si	N:P	C:Chla	α*	PQ α	Pm*	PQPm	E_c_	E_k_
11	450	Exp	0.75 ± 0.01	5.49	78.9	ND	14.4	33.2	1.62	1.56	374	1.45	16	217
11	130	Exp	0.78 ± 0.01	5.44	71.1	ND	13.1	20	1.02	1.14	213	1.28	18	207
11	40	Exp	0.45 ± 0.02	5.44	82.5	ND	15.1	16.9	1.5	1.17	251	1.22	11	176
11	20	Exp	0.35 ± 0.02	5.52	90.2	ND	16.3	22.3	1.64	1.23	225	1.15	3	148
7	450	Exp	0.67 ± 0.02	5.71	79.8	ND	14	42.6	1.19	1.06	356	1.25	32	263
7	130	Exp	0.65 ± 0.03	5.59	88.2	ND	15.8	27.1	1.23	1.16	221	1.26	4	157
7	40	Exp	0.57 ± 0.02	5.67	81.9	ND	14.5	21.5	1.85	1.35	329	1.46	6	149
7	20	Exp	0.43 ± 0.02	5.13	44.1	ND	8.6	18.5	1.78	1.22	273	1.24	4	154
3	450	Exp	0.55 ± 0.01	6.73	111	ND	16.5	47.6	0.88	1.09	197	1.22	26	183
3	130	Exp	0.51 ± 0.01	6.63	91.7	ND	13.8	42.4	1.55	1.2	233	1.29	13	171
3	40	Exp	0.44 ± 0.01	5.5	47.8	ND	8.7	19.1	1.42	1.22	211	1.62	3	169
3	20	Exp	0.34 ± 0.01	5.63	44.1	ND	7.8	16.2	1.28	1.02	170	1.3	11	255
11	130	N lim		20.3	297	9.9	14.6	101	0.59	1.14	67	1.24	17	150
7	450	N lim		24.1	357	11.5	14.8	137.2	0.99	1.26	181	1.64	5	207
7	130	N lim		27.5	265	11.9	9.6	102.7	0.88	1.17	117	1.5	4	164
7	40	N lim		22.7	224	9.6	9.9	63.7	0.78	0.93	121	1.59	17	239
3	130	N lim		18.5	201	ND	10.9	57	0.79	0.93	133	1.28	6	245
11	130	P lim		6.93	286	7.8	41.2	24.4	0.36	0.69	114	1.12	42	385
7	450	P lim		12	593	14.4	49.6	53.5	0.37	0.84	71	1.19	41	284
7	130	P lim		11.9	708	14.9	59.6	38.1	0.35	0.68	77	1.2	14	303
7	40	P lim		9.76	498	5	51.1	32.1	0.44	0.82	94	1.24	13	294
3	130	P lim		7.42	436	ND	58.8	21.6	0.33	0.6	94	1.2	10	369
11	130	Si lim		6.05	133	37.8	21.9	30.5	ND	ND	ND	ND	ND	ND
7	450	Si lim		6.6	205	43.3	31.1	30	ND	ND	ND	ND	ND	ND
7	130	Si lim		5.43	93.9	35.3	17.3	19	ND	ND	ND	ND	ND	ND
7	40	Si lim		5.98	164	38.9	27.5	16	ND	ND	ND	ND	ND	ND
3	130	Si lim		6.17	138	ND	22.5	16.8	ND	ND	ND	ND	ND	ND

The growth conditions depict acclimation to: Temperature (Temp) in °C, Light in μmol photons m^-2^ s^-1^ and the growth phase: exponential (Exp) or stationary growth under N (N lim), P (P lim) and Si (Si lim) limitation. Growth rates are d^-1^ ± Standard Error (S1 Fig). Samples at the stationary growth phase were taken after minimum 3 days with no increase in cell concentration. Growth curves up to the point of stationary growth phase harvesting are presented in S2, S3 and S4 Figs for N, P and Si limitation respectively. Stoichiometric rates are molar ratios except for C:Chl *a* which is the weight ratio. All the photosynthetic parameters are from O_2_ production. The maximum light utilization coefficient α* (the initial slope of the PE curve normalized to Chl *a*) is in mol O_2_ (mg Chl *a*)^-1^ h^-1^ (mol photons m^-2^ s^-1^)^-1^; the photosynthetic maximum, P_m_* is in μmol O_2_ (mg Chl *a*)^-1^ h^-1^; the photosynthetic quotient (PQ) is the ratio between O_2_ produced and C fixed, expressed at both α* and P_m_*; the compensation point, E_c_, and the light saturation parameter, E_k_, are both in μmol photons m^-2^ s^-1^. ND = not determined.

### Measurement of particulate organic matter

Chl *a* concentration was determined from duplicate, sub-samples filtered onto glass fiber filters (Whatman GF/F) and extracted in 10 ml of 94% ethanol for 24 h in darkness at room temperature [20]. Chl *a* was measured on a Cary, Varian Eclipse spectrofluorometer, calibrated with pure Chl *a* (Sigma). Duplicate filters were also prepared for determination of POC, PON, POP, and during the stationary growth phase also biogenic silicate (BSi). For POC, PON, and POP, acid-washed, pre-combusted GF/F filters were used, and BSi samples were filtrated onto 0.8 μm polycarbonate filters. The filters were allowed to dry and stored at room temperature (20°C) until determination of the element quantity. POC and PON were measured from the same filter with a mass spectrometer (Europa Scientific). POP was determined according to Solórzano and Sharp [21]. Filters for BSi determinations were digested using methods of Krausse et al. [22]. In brief, the filters were leached with NaOH in boiling water, neutralized with HCl, and analyzed directly for dissolved silicate (DSi) using standard colorimetric procedures [23].

### Measurement of PE relationship

Determination of the photosynthesis-irradiance (PE) relationship was conducted with both O_2_ production and ^14^C fixation. The same incubation time (30 min) was used for both methods. The PE incubator is a prototype constructed by B.G. Mitchell (Scripps Institute of Oceanography, USA). Briefly, the incubator has a rectangular shape (65 x 8 x 15 cm) with a halogen light source at one end, directed along a series of incubation chambers (16 light and 2 dark) spaced equidistant along the long axis of the incubator. Each chamber holds one 7 mL scintillation vial. Light passes through openings at the bottom of each chamber, the intensity regulated by the size of the opening. The incubator is water-cooled throughout.

Up to four PE incubators were used simultaneously; the same sample incubated in parallel for both O_2_ and ^14^C measurements. Cooling water was kept at the acclimated temperature of the culture. For each incubation, 2 dark and 12 light points were used for O_2_ determination and 2 dark and 16 light points were used for ^14^C uptake measurements. Irradiance ranged from 0 to ~2000 μmol photons m^-2^ s^-1^.

For O_2_ incubations, scintillation vials were filled completely (~7 mL) leaving no headspace. The O_2_ concentration was determined immediately before and after the incubation using a fiber optic oxygen sensor (PreSens GmbH, Fibox 3), calibrated against 0 and 100% air saturation of oxygen before each set of measurements (anoxic water created by adding sodium dithionite and oxygen saturated water by bubbling with air). Gross photosynthesis was calculated by adding the respiration, measured in the dark bottles, to net production.

Carbon incorporation was determined using the ^14^C isotope [24]. An activity of 0.73 kBq was added to 50 mL sample, which was subsequently distributed in scintillation vials (3 mL in each). After the incubation period (30 min), 200μL 1M HCl was added, and the scintillation vials were left open for 2 days, after which 4 mL Hi Safe scintillation liquid was added [25]. Radioactivity of the samples was determined directly from the incubation vials using a liquid scintillation counter (PerkinElmer Inc., Wallac Winspectral 1414). The amount of total dissolved inorganic carbon (DIC) was measured with a high-temperature combustion IR carbon analyzer (Unicarbo, Electro Dynamo). Primary production was calculated from the uptake of ^14^C knowing the total amount of added isotope and total DIC.

The PE relationship was examined by fitting the function of Platt et al. [26]:

$$P^{*}\;=\;P_{s}^{*}\left[1-\text{exp}\left(\frac{-\alpha \text{E}}{P_{s}^{*}}\right)\text{exp}\left(\frac{-\beta \text{E}}{P_{s}^{*}}\right)\right]$$ (1)

to the obtained data, where P_s_* is the maximum potential production in the absence of photoinhibition, production is measured in μmol C or O_2_ (mg Chl *a*)^-1^ h^-1^, E is irradiance in μmol photons m^-2^ s^-1^, α is the initial slope and *β* is the slope of the curve beyond the point of photoinhibition in mol C or O_2_ (mg Chl *a*)^-1^ h^-1^ (mol photons m^-2^ s^-1^)^-1^. At light saturation, the maximum photosynthetic rate, normalized to Chl *a* (P_m_*), is:

$$P_{m}^{*}=\;P_{s}^{*}\;{\left[\left(\frac{\alpha }{\alpha +\beta }\right)\left(\frac{\beta }{\alpha +\beta }\right)\right]}^{\left(\frac{\beta }{\alpha }\right)}$$ (2)

The photosynthetic quotient (PQ) was calculated by dividing the gross O_2_ production by the C fixation, which for this short incubation time was assumed to also represent gross production [11]. This was done for both the α* and P_m_* region of the PE curve, representing light limited and light saturated conditions respectively.

### Statistical treatment

The response surfaces were modelled using the Natural Neighbor algorithm in Surfer 12 (Golden Software). To compare the goodness of fit we calculated the coefficient of multiple determination (R^2^) from the total sum of squares (SStot) and the residual SS from the model (SSres) according to the equation:

$$R^{2}\;=1-\;\left(\frac{SS_{res}}{SS_{tot}}\right)$$ (3)

In addition to the response surface, we fitted a plane to the same data, using the polynomial regression option in Surfer. The two models can be compared with the SSres. A well-fitting model would yield a smaller SSres and consequently have lower residual variance than a poor-fitting model. To test for statistical difference between models we used Fisher’s F test of variance.

The experimental data was not replicated for individual combinations of light and temperature and we are not able to evaluate the variability for specific combinations. However, by pooling data into e.g. temperature, the variability within a given temperature, within a range of light acclimation (20–450 μmol photons m^-2^ s^-1^), can be estimated. In order to test for difference in variance between groups (>2) we used Levene’s test.

Mean observations were compared either with Student’s t-test for comparing 2 groups or Analysis of Variance (ANOVA) when comparing 3 groups. Tukey’s Post Hoc test was in the latter case used to make pairwise comparisons of groups.

## Results

### Growth and stoichiometry

**Fig 1:**
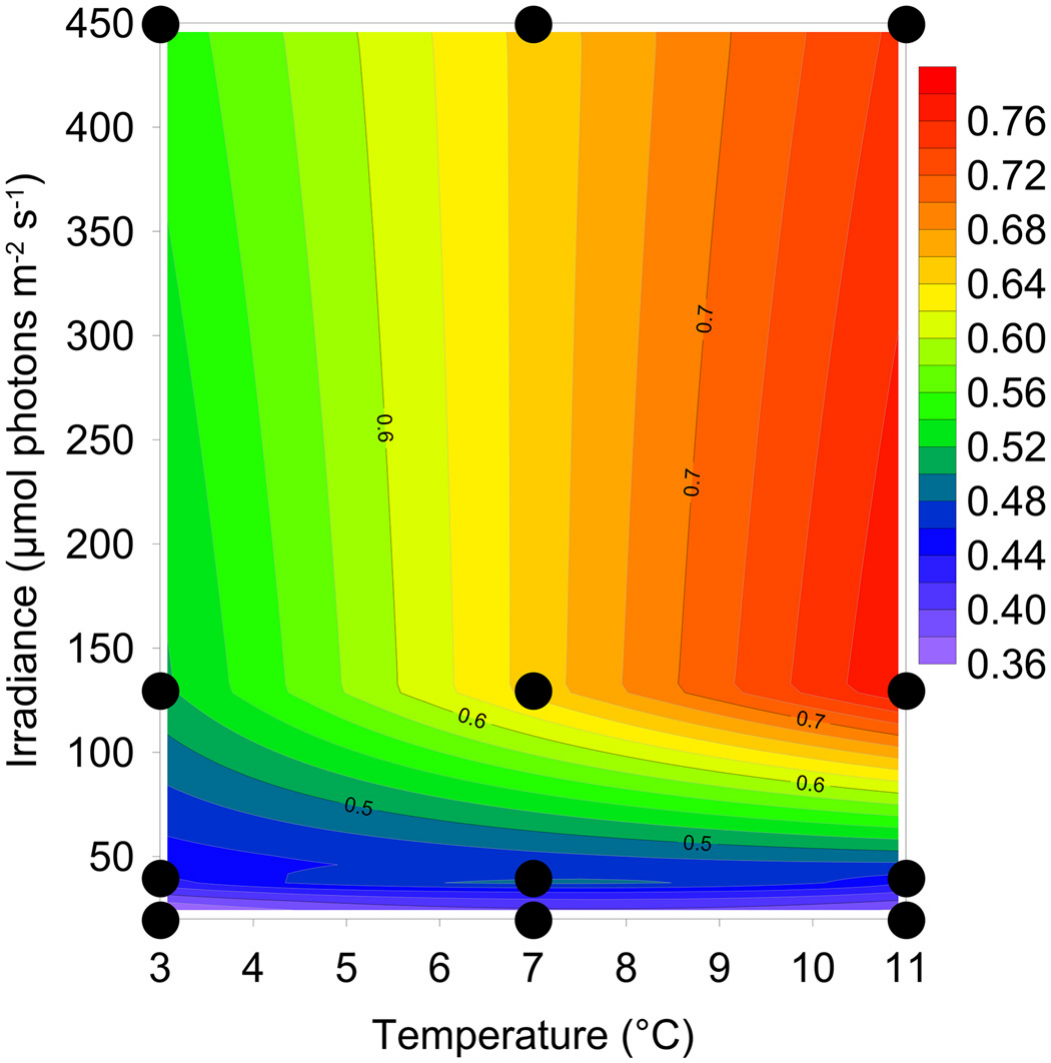
The growth rate during exponential growth of *Chaetoceros wighamii* acclimated to different irradiance and temperature. The response surface represents the model fit to the data in Table 1, and a statistical comparison with a fitted plane is presented in Table 2. Dots represent the different combinations of light and temperature.

There was clear interaction effect of light and temperature on growth and stoichiometry (Fig 1, Tables 1 and 2). The maximum growth rate was observed at 11°C in high light (~0.8 d^-1^) and at 3°C the growth rate was ~30% lower under similar light conditions. The culture did not grow at 15°C, and 11°C is apparently close to the maximum temperature allowing growth for this cold-water species. Growth was approximately equal at the highest irradiances (0.78 and 0.75d^-1^ at 130 and 450 μmol photons m^-2^ s^-1^, respectively), but clearly lower at light <130 μmol photons m^-2^ s^-1^. At 20 μmol photons m^-2^ s^-1^ the growth rate was ~50% of the maximum growth rate, at similar growth temperature.

**Table 2:** The coefficient of multiple determination (R^2^) for a fitted plane and the modeled response surface with a statistical test of differences between these two ways of representing the data.

	Growth rate	Stoichiometry				Photosynthetic parameters (O_2_)			
	μ	C:N	C:P	N:P	C:Chla	α*	Pm*	E_c_	E_k_
Total SS	0.237	2.519	4770	105.1	1397	0.98	46758	976	17368
Residual SS:									
Fitted plane	0.119	1.236	3524	68.7	333	0.71	28667	301	13005
Modeled response surface	0.009	0.174	201	7.8	18	0.09	5430	96	1074
R^2^									
Fitted plane	0.50	0.51	0.26	0.35	0.76	0.27	0.39	0.69	0.25
Modeled response surface	0.96	0.93	0.96	0.93	0.99	0.91	0.88	0.90	0.94
p-value	<0.001	0.003	<0.001	<0.001	<0.001	0.001	0.007	0.05	<0.001

**Fig 2:**
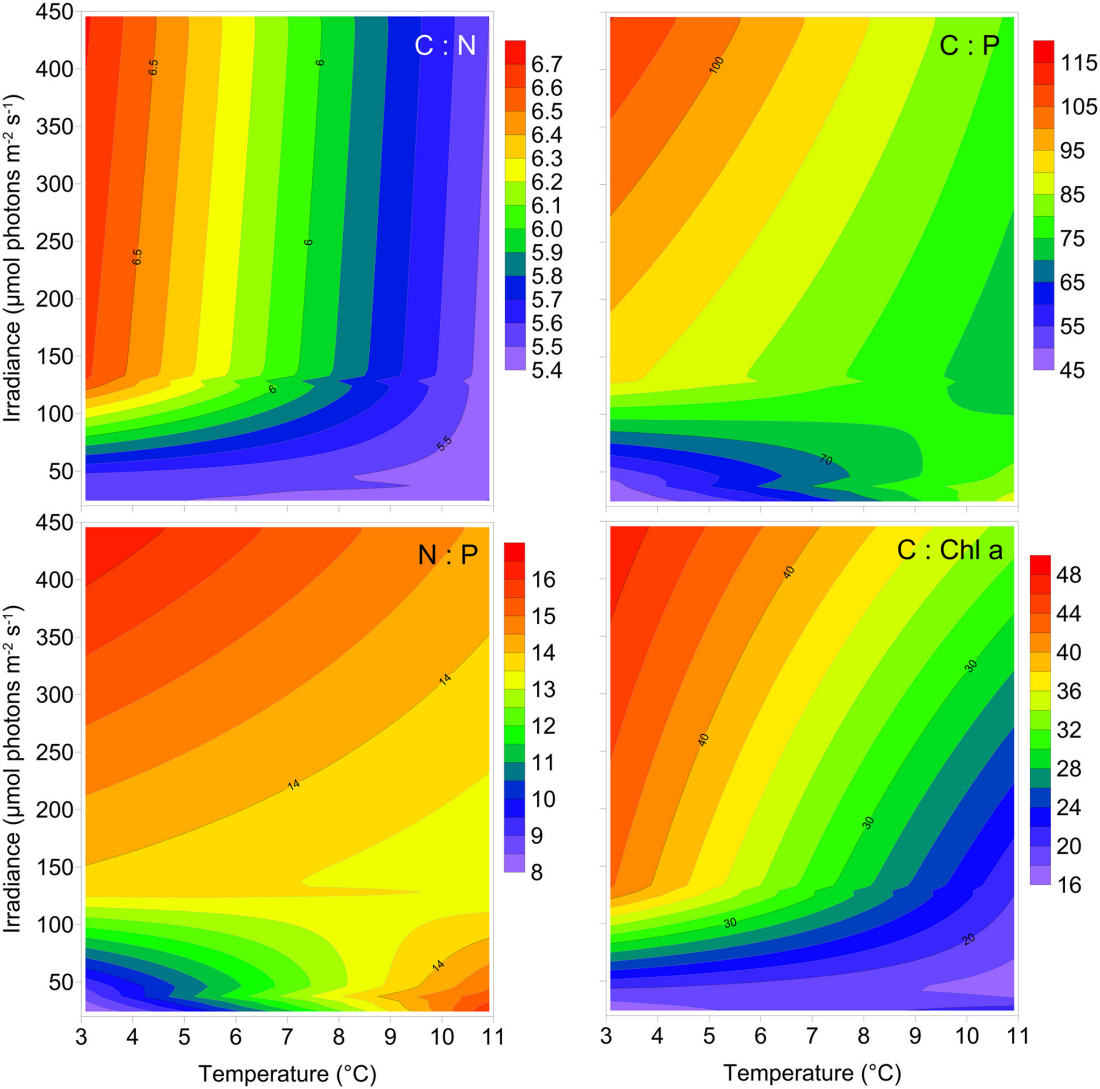
The stoichiometric relationships: C:N (molar ratio), C:P (molar ratio), N:P (molar ratio) and C:Chl *a* (weight ratio) during exponential growth of *Chaetoceros wighamii* acclimated to different irradiance and temperature. The data is presented in Table 1, and a statistical comparison with a fitted plane is presented in Table 2.

The R^2^ is a measure of the goodness of fit and was calculated from the total and residual sum of squares (SS) according to Eq 3. The fitted plane represents a plane tilted to best fit the data (by polynomial regression), whereas the modeled response surface is presented in Figs 1, 2 and 5. The p-values are from Fishers-F test of variance comparing the residuals from the fitted plane with the modeled response surface.

During exponential growth, the C:N and C:P ratios were clearly affected by both light and temperature, containing relatively more C at combinations of high light and low temperature (Fig 2). The average C:N ratio was 5.71 ± 0.48 (SD, n = 12) and the average C:P ratio was 75.93 ± 20.82 (SD, n = 12). The average N:P ratio was 13.2 ± 3.1 (SD, n = 12) and decreased slightly with increasing growth rate, except at low temperature and low light where the N:P ratio was clearly lower than in other treatments (Table 1, Fig 2). The C:Chl *a* ratio was also affected by both light and temperature during exponential growth. The lowest ratio was found at the lowest light and temperature, and at 3°C there was a clear increase in the C:Chl *a* ratio with increasing light (Fig 2). At the highest temperature (11°C) the effect of light acclimation on C:Chl *a* ratio was less pronounced (Table 1, Fig 2).

Statistical comparisons of the modeled response surfaces, for growth and stoichiometric ratios, compared with modeled flat planes during exponential growth are presented in Table 2. The residual sum of squares for the response surface was lower than the plane, and a better fit to the data (p <0.01; Table 2).

**Fig 3:**
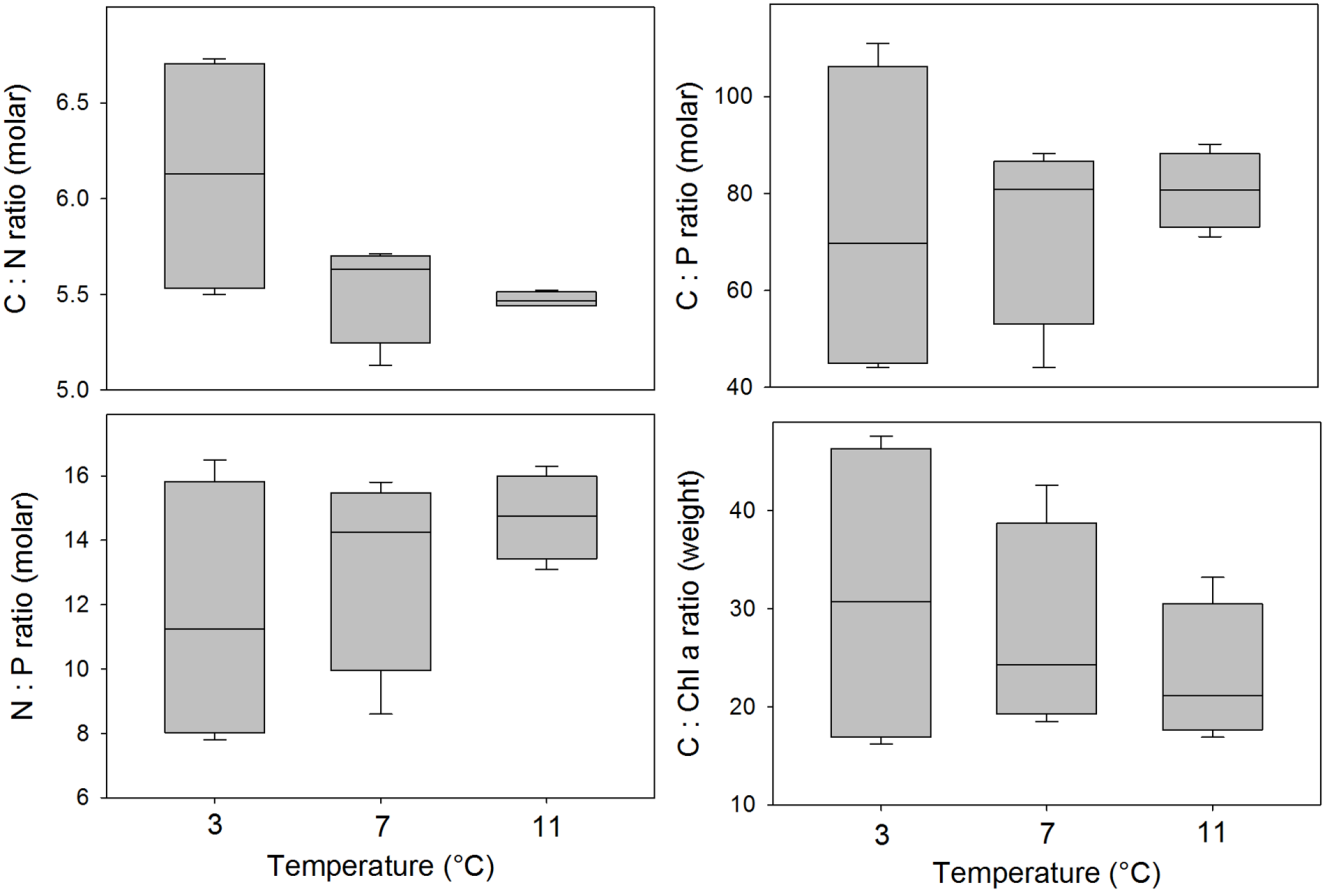
The stoichiometric relationships: C:N (molar ratio), C:P (molar ratio), N:P (molar ratio) and C:Chl *a* (weight ratio) during exponential growth of *Chaetoceros wighamii* acclimated to different temperature. The culture was acclimated to four irradiance levels for each temperature (20, 40, 130 and 450 μmol photons m^-2^ s^-1^). The horizontal line is the median, the box represents the 25–75% confidence interval, and the error bars the 10–90% confidence interval (n = 4). There were no statistical difference between means, but there was an effect of temperature on the variance (see text for details). The data can be found in Table 1.

The range in the stoichiometric data was clearly higher at low temperature during exponential growth (Fig 3). There was little to no evidence that the temperature had an effect on stoichiometry when comparing means statistically (ANOVA: C:N, p = 0.09; C:P, p = 0.88, N:P, p = 0.43; C:Chl *a*, p = 0.63), but the variability measured as standard deviation was for C:N, C:P, N:P and C:Chl *a* ratios a factor 16.4, 1,7, 2.5 and 2.3 higher at 3°C compared with 11°C. Testing for difference in variance statistically, yielded some evidence for differences between temperatures (Levene’s test: C:N, p <0.001; C:P, p = 0.01, N:P, p = 0.06; C:Chl *a*, p = 0.06). The apparent higher variability at low temperature was caused by a much wider spread between the low and high light acclimated cultures, where high light elevated all the ratios (Table 1).

**Fig 4:**
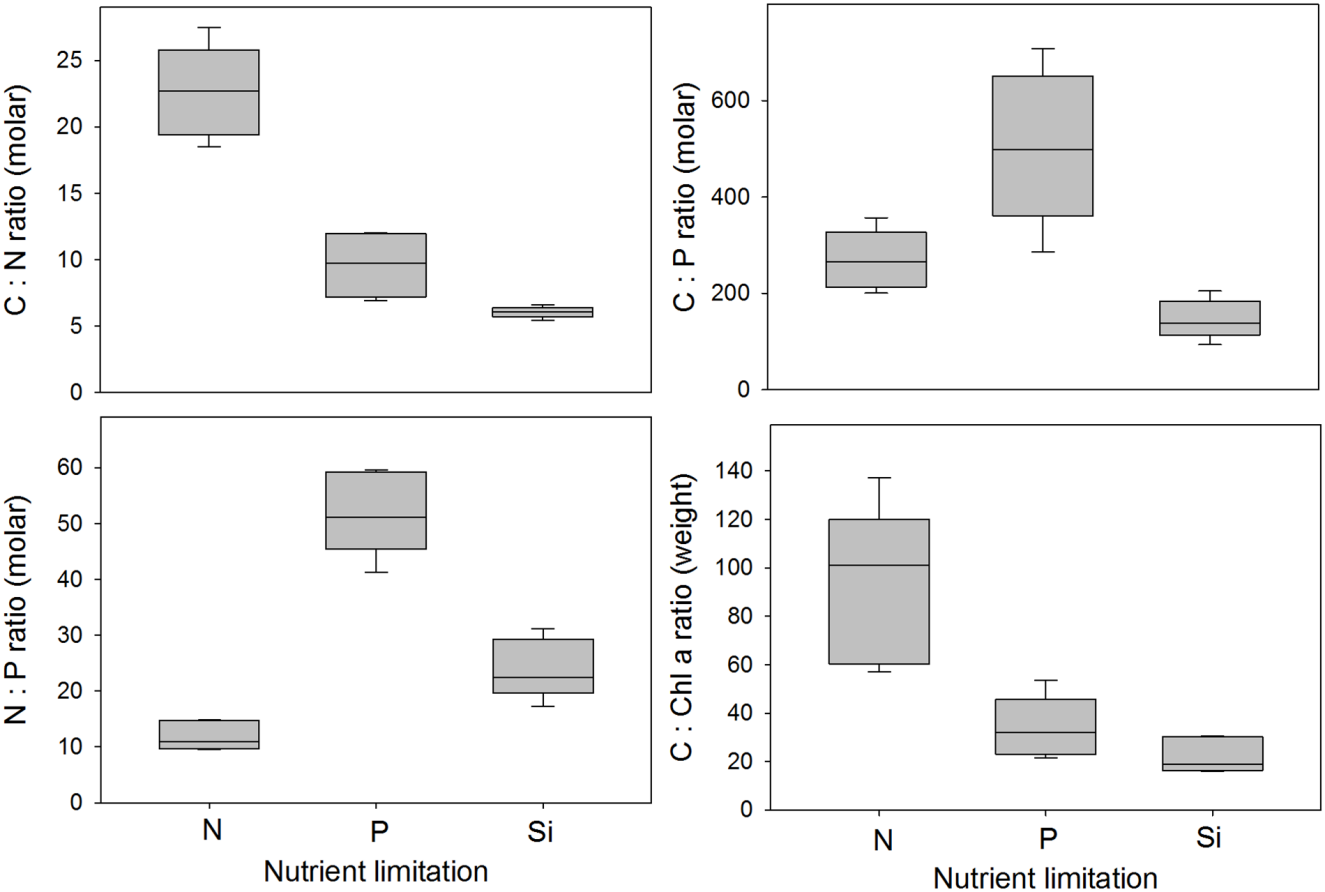
The stoichiometric relationships: C:N (molar ratio), C:P (molar ratio), N:P (molar ratio) and C:Chl *a* (weight ratio) of *Chaetoceros wighamii* during different nutrient limitation and combination of growth light and temperature (details and data can be found in Table 1). The horizontal line is the median, the box represents the 25–75% confidence interval, and the error bars the 10–90% confidence interval (n = 5). Statistical comparisons between the nutrient limitations are presented in Table 3.

During stationary growth, the stoichiometry depended on the nutrient limitation (Fig 4, Table 3). During N limitation, the average stoichiometric C:N and C:P ratios increased by a factor of 4.0 and 3.5, respectively. During P limitation, the average C:P increased by a factor of 6.6; whereas the average C:N ratio increased only 1.7 fold. During Si limitation, the C:N was comparable to the exponential growth phase, but C:P increased 2-fold. The N:P ratio was mostly affected by P limitation: under P limitation the average N:P ratio increased ~4 fold, under Si limitation the N:P ratio increased 1.8-fold whereas under N limitation the N:P decreased by 9% compared with the average N:P ratio during exponential growth. The average C:Chl *a* ratio increased by factors of 2.3, 2.2 and 1.1 under N, P and Si limitation, respectively. During Si limitation the C:Si increased 3.6-fold compared with N or P limitation. Statistical comparisons of the effect of the different nutrient limitations on the stoichiometric ratios are presented in Table 3.

**Table 3:** ANOVA table comparing the effect of nutrient limitations (N, P or Si) on stoichiometric ratios (Fig 4) with Tukey’s Post Hoc test for pairwise comparison.

C:N ratio					
Source of Variation	DF	SS	MS	F	p
Between Groups	2	761	381	63.4	<0.001
Residual	12	72.0	6.00		
Total	14	833			
**Comparison**	**Diff of Means**		**q**	**p**	
N to Si limitation	16.6		15.1	<0.001	
N to P limitation	13.0		11.9	<0.001	
P to Si limitation	3.6		3.25	0.095	
**C:P ratio**					
**Source of Variation**	**DF**	**SS**	**MS**	**F**	**p**
Between Groups	2	330085	165043	16.0	<0.001
Residual	12	123663	10305		
Total	14	453748			
**Comparison**	**Diff of Means**		**Q**	**P**	
P to Si limitation	357		7.87	<0.001	
N to P limitation	235		5.19	0.009	
N to Si limitation	122		2.69	0.181	
**N:P ratio**					
**Source of Variation**	**DF**	**SS**	**MS**	**F**	**p**
Between Groups	2	4231	2115	69.1	<0.001
Residual	12	367	30.6		
Total	14	4598			
**Comparison**	**Diff of Means**		**Q**	**P**	
N to P limitation	40.1		16.2	<0.001	
P to Si limitation	28.0		11.3	<0.001	
N to Si limitation	12.1		4.89	0.012	
**C:Chl *a* ratio**					
**Source of Variation**	**DF**	**SS**	**MS**	**F**	**p**
Between Groups	2	14034	7017	16.5	<0.001
Residual	12	5118	426		
Total	14	19152			
**Comparison**	**Diff of Means**		**Q**	**p**	
N to Si limitation	69.9		7.56	<0.001	
N to P limitation	58.4		6.32	0.002	
P to Si limitation	11.5		1.24	0.663	

### Photosynthetic properties

**Fig 5:**
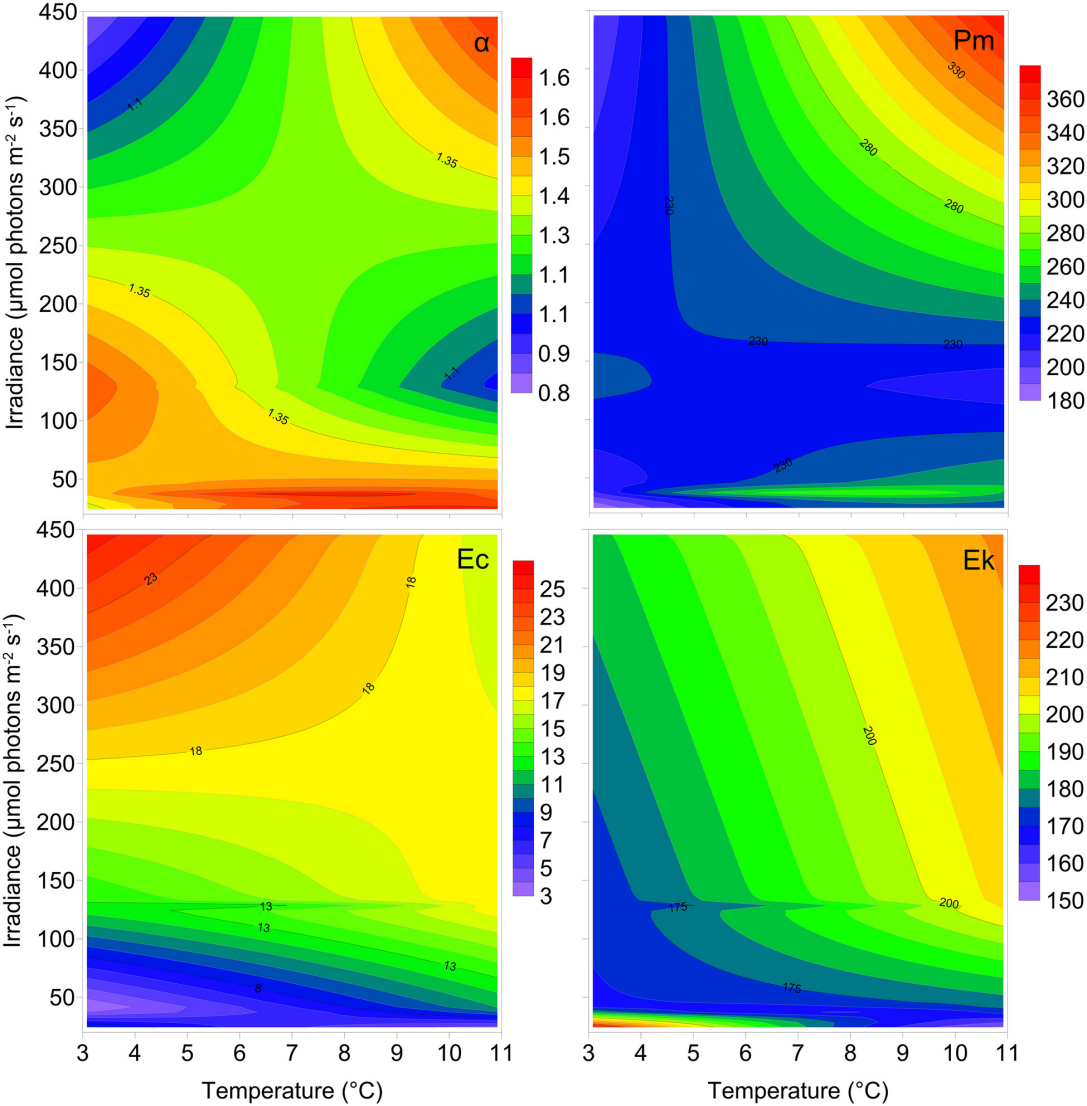
The maximum light utilization coefficient (α*) in mol O_2_ (mg Chl *a*)^-1^ h^-1^ (mol photons m^-2^ s^-1^)^-1^, photosynthetic maximum (Pm*) in μmol O_2_ (mg Chl *a*)^-1^ h^-1^, light compensation point (Ec) in μmol photons m^-2^ s^-1^ and light saturation parameter (Ek) in μmol photons m^-2^ s^-1^ during exponential growth of *Chaetoceros wighamii* acclimated to different irradiance and temperature. The data is presented in Table 1, and a statistical comparison with a fitted plane is presented in Table 2.

During exponential growth, there was interaction effect of light and temperature on photosynthetic properties (Fig 5, Tables 1 and 2). The Chl *a*-normalized initial slope of the PE curve, also termed maximum light utilization coefficient (α*), was on average 1.41 ± 0.30 (SD, n = 12) mol O_2_ (mg Chl *a*) ^-1^ h^-1^ (mol photons m^-2^ s^-1^)^-1^ (Fig 3, Table 1). The average maximum photosynthetic production (P_m_*) was 254 ± 65 (SD, n = 12) μmol O_2_ (mg Chl *a*)^-1^ h^-1^ and highest for the high light and high temperature acclimated culture (Fig 5). The average compensation point, E_c_, was 12.2 ± 9.5 (SD, n = 12) μmol photons m^-2^ s^-1^ and the light saturation parameter, E_k_, was 188 ± 40 (SD, n = 12) μmol photons m^-2^ s^-1^.

Statistical comparisons of the modeled response surfaces for the photosynthetic parameters, compared with modeled flat planes during exponential growth are presented in Table 2. The residual sum of squares for the response surface was lower than the plane, and for α*, P_m_* and E_k_ clearly better fit to the data (p <0.01; Table 2). The statistical comparison for Ec was not as clear when comparing sum of squares, and yielded a probability value of 0.05 (Table 2).

The rate of oxygen production to carbon fixation or photosynthetic quotient (PQ) was 1.2 ± 0.14 (SD, n = 12) at the α* region of the PE curve and 1.3 ± 0.13 (SD, n = 12) at the P_m_* region during exponential growth (Table 1). Comparing the two means statistically, yielded a probability value of 0.07 (Student’s t-test, n = 12). The compensation point for primary production E_c_ and the light saturation parameter E_k_ was in general lowest for the low light acclimated cultures (Fig 5).

At the stationary growth phase, α* and P_m_* decreased whereas E_c_ and E_k_ increased compared with the exponential growth phase, but was more affected by P than N limitation (Table 1). The average reduction of α* and P_m_* was 43% and 51% respectively during N limitation; during P limitation the reduction was 74% and 65% respectively. The average E_c_ increased by a factor 1.22 and 1.96 during N and P limitation respectively, and E_k_ increased by a factor 1.07 and 1.74 during N and P limitation respectively.

**Fig 6:**
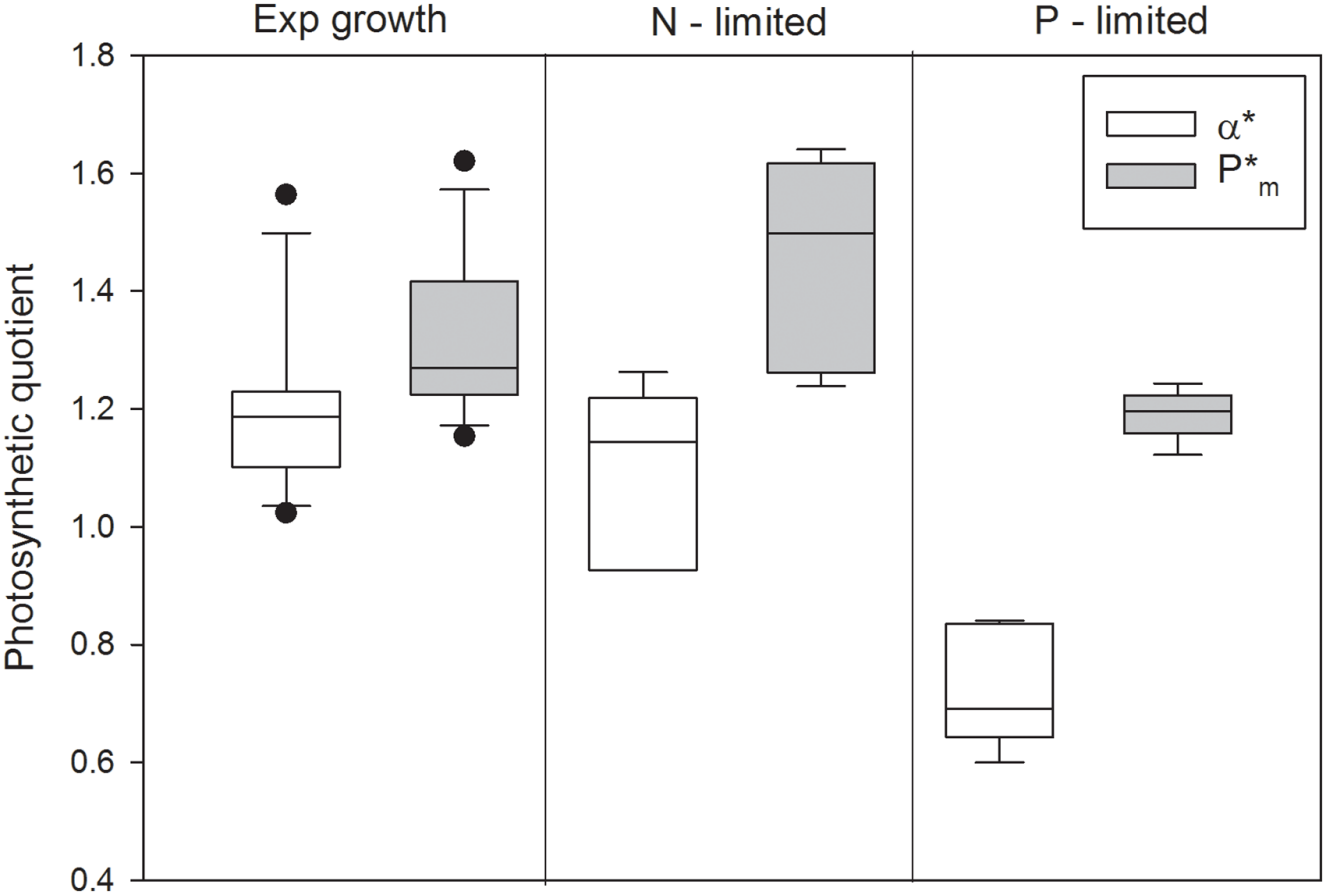
The photosynthetic quotient (PQ; mol O_2_ produced per mol C fixed) at exponential and stationary growth phases (both N and P limited), and at the initial slope (α*) and photosynthetic maximum (Pm*) of the PE curve. The horizontal line is the median, the box represents the 25–75% confidence interval, and the error bars the 10–90% confidence interval (n = 12 for exponential growth; n = 5 for N and P limitation). The data is presented in Table 1.

During P and N limited growth the PQ values were 0.7 ± 0.10 (SD, n = 5) and 1.1 ± 0.15 (SD, n = 5) at α* and 1.2 ± 0.04 (SD, n = 5) and 1.5 ± 0.18 (SD, n = 5) at P_m_*, respectively for P and N limitation (Fig 6), and there was strong statistical support for the PQ in the α* and P_m_* regions of the PE curve being different (Student’s t-test: p <0.001 for P limited growth and p = 0.009 for N limited growth, n = 5).

## Discussion

### Interaction effects and variability

The better fit of the response surface compared with the fitted plane can be interpreted as second-order effects, and is an indication of interaction effects. Without any interaction effects, the response surface would be equal along the non-affecting parameter e.g. similar response to light acclimation regardless of temperature (or vice versa). The visual representation of the data clearly shows interaction effects for growth and the stoichiometric parameters during exponential growth, and additionally for α*, P_m_* and E_k_. For E_c_ the difference between the response surface and the flat plane was less pronounced (but with p = 0.05), and here the graphical representation indicates that light acclimation has the most effect on E_c_, indicating more uncertainty about the presence of an interaction effect for this parameter.

The lack of replication in our experimental set-up prevents estimation of variability for single combinations of light and temperature. However, pooling the data by temperature enables estimation of variability over a range of light acclimations. For the stoichiometric parameters, the data suggests that variability, measured as variance, is dependent on temperature, and it is higher at low temperature. In particular for the C:N and C:P ratios where the p-value was ≤ 0.01. For the N:P and C:Chl *a* ratios, the p-value was 0.06 leaving more uncertainty in the interpretation, but viewing the overall results, the possibility of increasing variability with lower temperature for the N:P and C:Chl *a* ratios can at least not be excluded.

### Growth and stoichiometry

The interplay between environmental factors such as light, temperature and nutrient availability and the physiology of the cell determines the growth rate and stoichiometric composition of the major elements in algae. Traditionally factors such as the growth-limiting nutrient have been used to model nutrient uptake and growth [27], and recent advances have started to incorporate uptake-protein regulation into this equation [28–29]. The latter is an important step as it incorporates the nutrient history of the primary producers, which is decisive in regulating the uptake rate determined by e.g. the number of uptake sites. The present data support the growing understanding of the interaction between fundamental abiotic parameters that should be included in growth models.

Geider and La Roche [30] pointed out in their review on algal stoichiometry that relatively few studies examine the phenotypic flexibility in C:N:P during exponential growth and that more studies are needed in order to better understand the effect of temperature and light on these ratios. Under nutrient replete conditions the variability in the C:N:P ratio is normally larger between different species than between different environmental conditions such as variation in temperature [30]. Our data support this to some extent, as there was low variability in the C:N during active growth. However, C:P and N:P was twofold different between the lowest and highest value. The larger variability in C:P and N:P was caused by high P content relative to C and N at low light and temperature acclimation. Strong latitudinal patterns in the C:N:P ratio was recently described, and a lower than average ratio was associated with high latitudes [31]. Martiny et al. [31] suggested this lower C:N:P ratio to be caused by the largely diatom dominated communities present in cold water, but diatoms have also been associated with higher C:N:P ratio [32]. Our results here imply that there is a temperature effect, with lower C:N:P in low temperature and light.

The intracellular concentration of P is known to be influenced by the concentration of P-rich ribosomes with their associated rRNA [2]. Increasing rRNA, coupled with increasing growth rates, have been shown to decrease the N:P ratio over a range of different organisms and biotopes [33]. Hillebrand et al. [34] found a similar trend of decreasing N:P ratio and variability with increasing growth rate in phytoplankton, suggesting that fast-growing phytoplankton in general require more P, and also have a more confined N:P ratio compared with slow-growing phytoplankton. Recently, temperature was also shown to affect the concentration of ribosomes in phytoplankton; at a constant protein synthesis, relatively more ribosomes are needed at low temperature [35]. This is in line with the temperature effect that we observed, and the most plausible reason for the reduced C:P and N:P at low temperature and light.

Once one or more nutrients are depleted, the stoichiometry has in general a much wider window of variability [2]. Typically, the ratio of C:N:P increase as C fixation continues for some time after cells have stopped dividing, which is supported by our observations. In particular diatoms are known to increase the C:N:P during stationary growth, and the extra carbon can have implications for the biogeochemical flux of carbon in the system [32]. The excess carbon can be stored as an energy reserve such as lipids [36]. Surplus N can be stored as protein, free amino acids or put into photosynthetic pigments [2,37], and P can be stored as polyphosphate [38]. During stationary growth, P limitation had the most pronounced effect on the N:P ratio, as opposed to N and Si limitation, suggesting that P content per biomass unit is less flexible than the N content in *C*. *wighamii*. This was also supported by increasing N:P during Si limitation. The much increased N:P at P and Si limitation suggests active uptake and storage of 2–4 fold the concentration of N during stationary growth phase, relative to P.

The C:Chl *a* ratio was, as expected, strongly influenced by light acclimation, as the cells acclimate to low light conditions by increasing photosynthetic pigmentation [39]. The data suggests a second order temperature effect, with the effect of light acclimation becomes much greater at the lowest temperature. There is little evidence to suggest temperature effects on C:Chl *a* ratio [40], but similar results were found in the cold water diatom *Skeletonema costatum* which had higher variability in the Chl *a* content per cell at low temperatures [41]. During the stationary growth phase, the C:Chl *a* ratio was ~4 fold higher during N limitation than during P or Si limitation, which can be attributed to the fact that Chl *a* contains N but not P or Si [2].

### Primary production and the photosynthetic quotient

Light and temperature have several well-known effects on primary production [42]. Under natural conditions with fluctuating light intensity there will be a continuous acclimation of light absorption and photosynthetic activity through the production of photosynthetic pigments and regulation of the energy channeled to the photochemical reaction centers. The light reactions are not directly dependent on temperature, but temperature affects enzymatic processes, membrane fluidity and intermolecular collision processes [42]. Light acclimation will affect photosynthesis under both limiting and saturating light conditions, whereas temperature will mostly affect photosynthesis at saturating light conditions [42]. As such, photosynthetic production will be optimized under all but the most limiting environmental conditions. The present experiments support these basic paradigms also for the cold-water diatom, despite (or more accurately: by means of) the observed variability in stoichiometry.

The growth rate and photosynthetic properties (α*, P_m_*, E_c_ and E_k_) we observed for *C*. *wighamii* were similar to published values [43], but expanded on these by including the interaction effect between light and temperature, and also including different nutrient limitation. Generally, the decreasing α* and P_m_* and increasing E_c_ and E_k_ can be expected when the cells stated to experience nutrient stress and optimizing photosynthetic production becomes less important. An apparent paradox was the difference between N and P limitation, where P limitation seemingly affect α* and P_m_* more than N limitation. Photosynthetic pigments contain N but no P, so intuitively this seems like a contradiction. However, both of these photosynthetic parameters were normalized to Chl *a*, which is the norm in the literature [11], and normalizing to POC instead yields an opposite result where α and P_m_ under N limitation are approximately half of that under P limitation. This highlight the importance of considering the biomass currency used to compare data.

The most surprising finding was the low (<1) PQ values at nutrient and light limitation. For *Pycnococcus provasolii* it has been shown that the PQ was affected by both light acclimation and incubation light intensity [44]; where decreasing growth light decreased the PQ. The lowest PQ recorded by Iriarte [44] was 0.7, and this low value was suggested to be caused by an underestimation of O_2_ production due to photorespiration. Photorespiration is a net loss process where O_2_ replaces CO_2_ at the rubisco enzyme catalyzing the carbon fixation, resulting in consumption of 3 O_2_ for every CO_2_ produced [45]. Photorespiration may serve a function, such as a protective mechanism to avoid reactive oxygen species during photosynthesis [46], or in the assimilation of nitrate [47].

Photorespiration alone cannot explain PQ values <0.75, but unbalanced growth, where respiration affects the ratio between produced O_2_ and fixed C, can [14]. Photorespiration and unbalanced growth are the most plausible reason why PQ values <1 were observed in our work and it is interesting that this would occur at low incubation light and nutrient limitation. All PQ ratios <1.0 were recorded under nutrient stress (all P limitation and two N limitation treatments). If photorespiration has any function in enhancing nutrient uptake, the energy deficit to assimilate nutrients was overcome when the incubation conditions were at P_m_, an aspect that is deserving of further study.

## Conclusion

There were clear interaction effects between light and temperature on growth, stoichiometric composition and photosynthetic parameters of *C*. *wighamii*. Getting a grip on these dynamics will improve our capabilities to model primary production and biomass concentration in the ocean based on satellite images and environmental variability. The present data suggests that several key parameters in stoichiometry are more variable at low temperature. The C:N:P uptake ratio and stoichiometry of phytoplankton is important as it directly affects biogeochemical cycling of key nutrients. The large variability at low temperature suggests that it is particularly challenging to accurately model this in cold-water (e.g. Arctic and Sub-Arctic) regions under ongoing climate change.

## Supporting Information

S1 Fig Exponential growth curves. Increase in cell numbers during the exponential growth phase for the different combinations of light and temperature acclimation. The line represents the linear fit to the natural logarithm (ln) transformed cells mL^-1^, and the slope is the growth rate d^-1^. The dotted lines represent the 95% confidence intervals.
(TIF)

S2 Fig N-limited growth curves. Increase in cell numbers during N-limitation until the point of harvesting for the different combinations of light and temperature acclimation. The y-axis is the natural logarithm (ln) transformed cells mL^-1^.
(TIF)

S3 Fig P-limited growth curves. Increase in cell numbers during P-limitation until the point of harvesting for the different combinations of light and temperature acclimation. The y-axis is the natural logarithm (ln) transformed cells mL^-1^.
(TIF)

S4 Fig Si-limited growth curves. Increase in cell numbers during Si-limitation until the point of harvesting for the different combinations of light and temperature acclimation. The y-axis is the natural logarithm (ln) transformed cells mL^-1^.
(TIF)
