# Supplementary figures and images for: Interaction Effects of Light, Temperature and Nutrient Limitations (N, P and Si) on Growth, Stoichiometry and Photosynthetic Parameters of the Cold-Water Diatom Chaetoceros wighamii
Source: PLoS One. 2015 May 20;10(5):e0126308. doi: 10.1371/journal.pone.0126308 (PMC4438981; doi:10.1371/journal.pone.0126308)

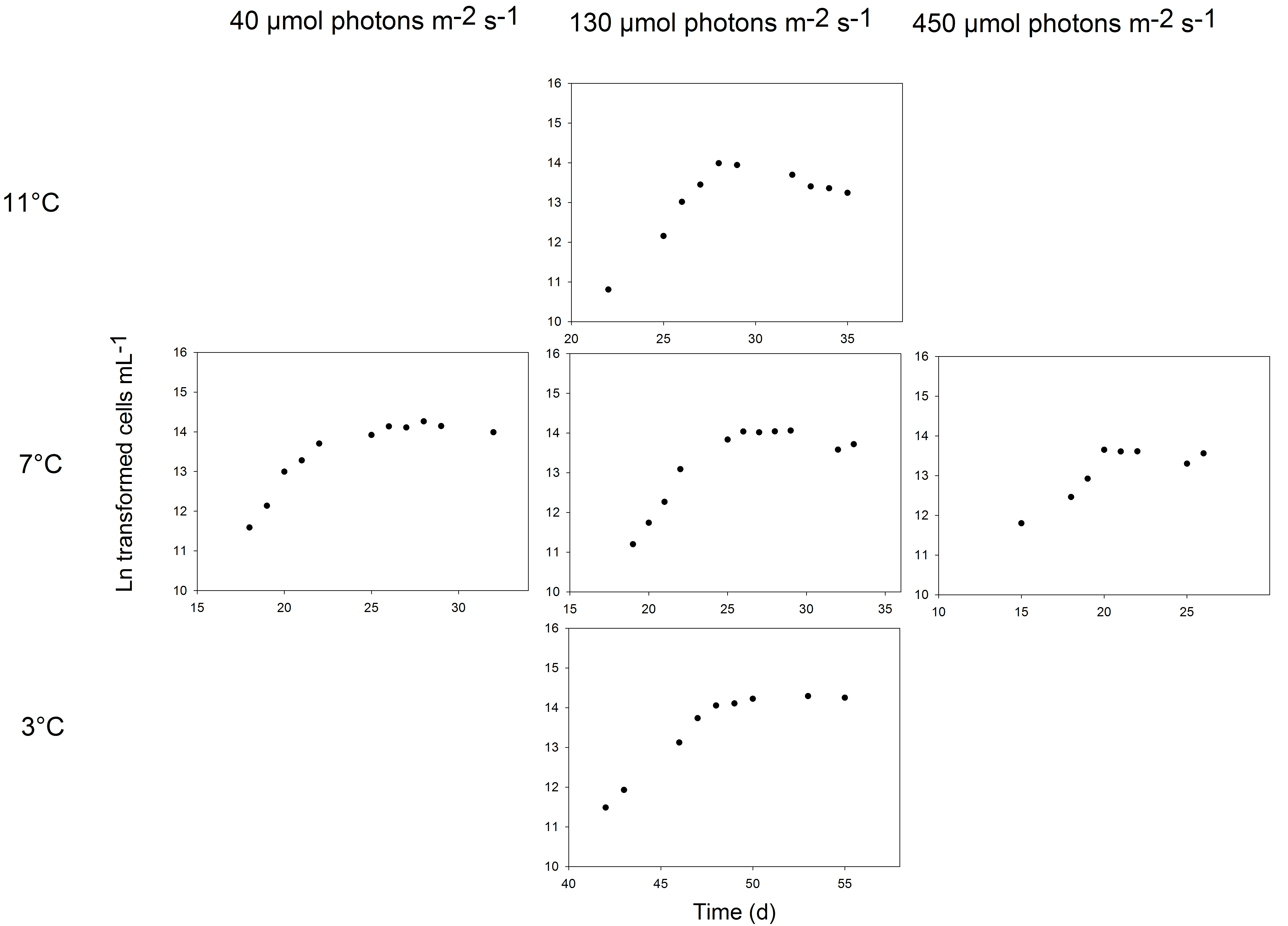

Supplement: S2 Fig — Increase in cell numbers during N-limitation until the point of harvesting for the different combinations of light and temperature acclimation. The y-axis is the natural logarithm (ln) transformed cells mL-1. (TIF) [file pone.0126308.s002.tif]

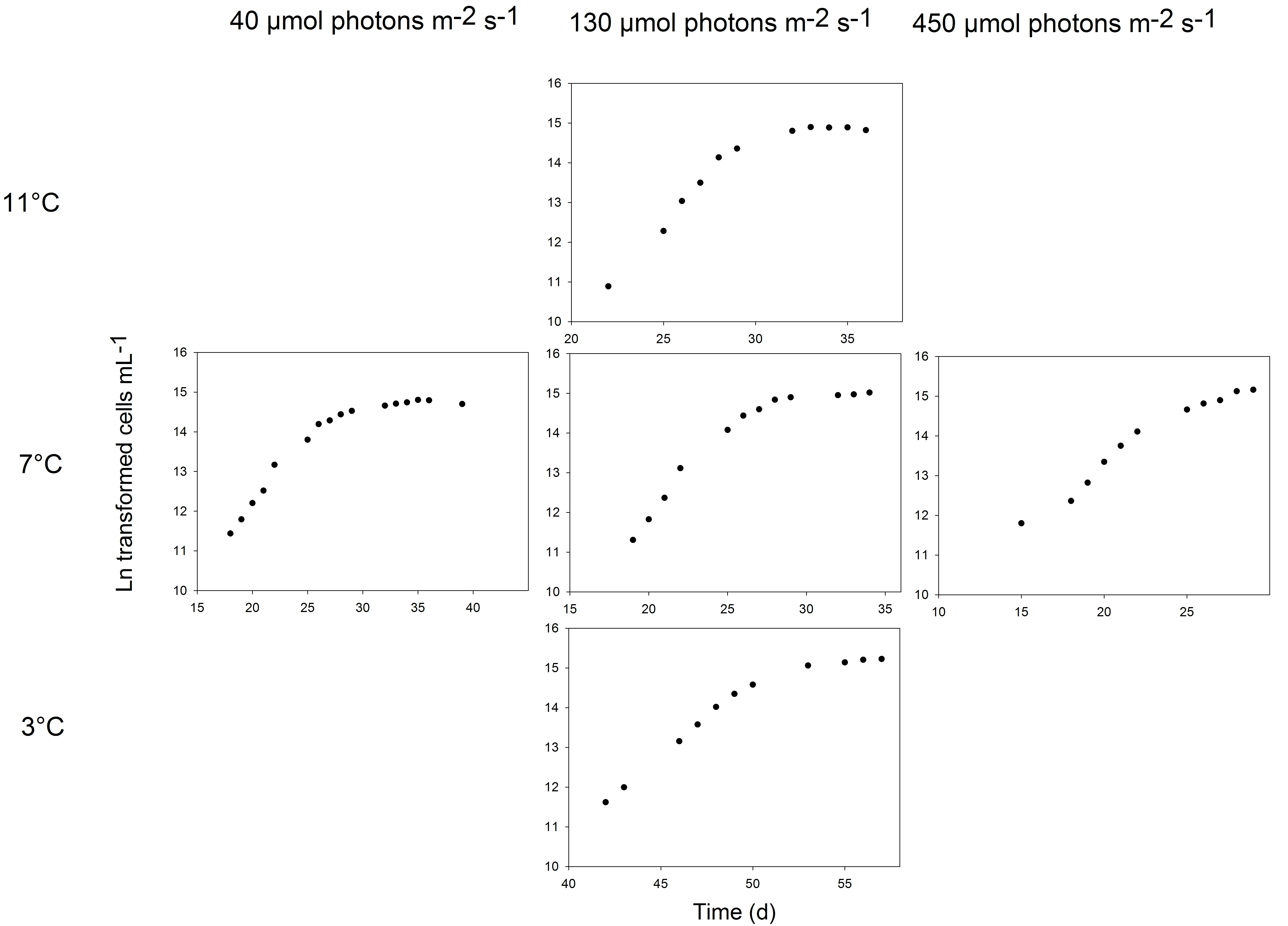

Supplement: S3 Fig — Increase in cell numbers during P-limitation until the point of harvesting for the different combinations of light and temperature acclimation. The y-axis is the natural logarithm (ln) transformed cells mL-1. (TIF) [file pone.0126308.s003.tif]

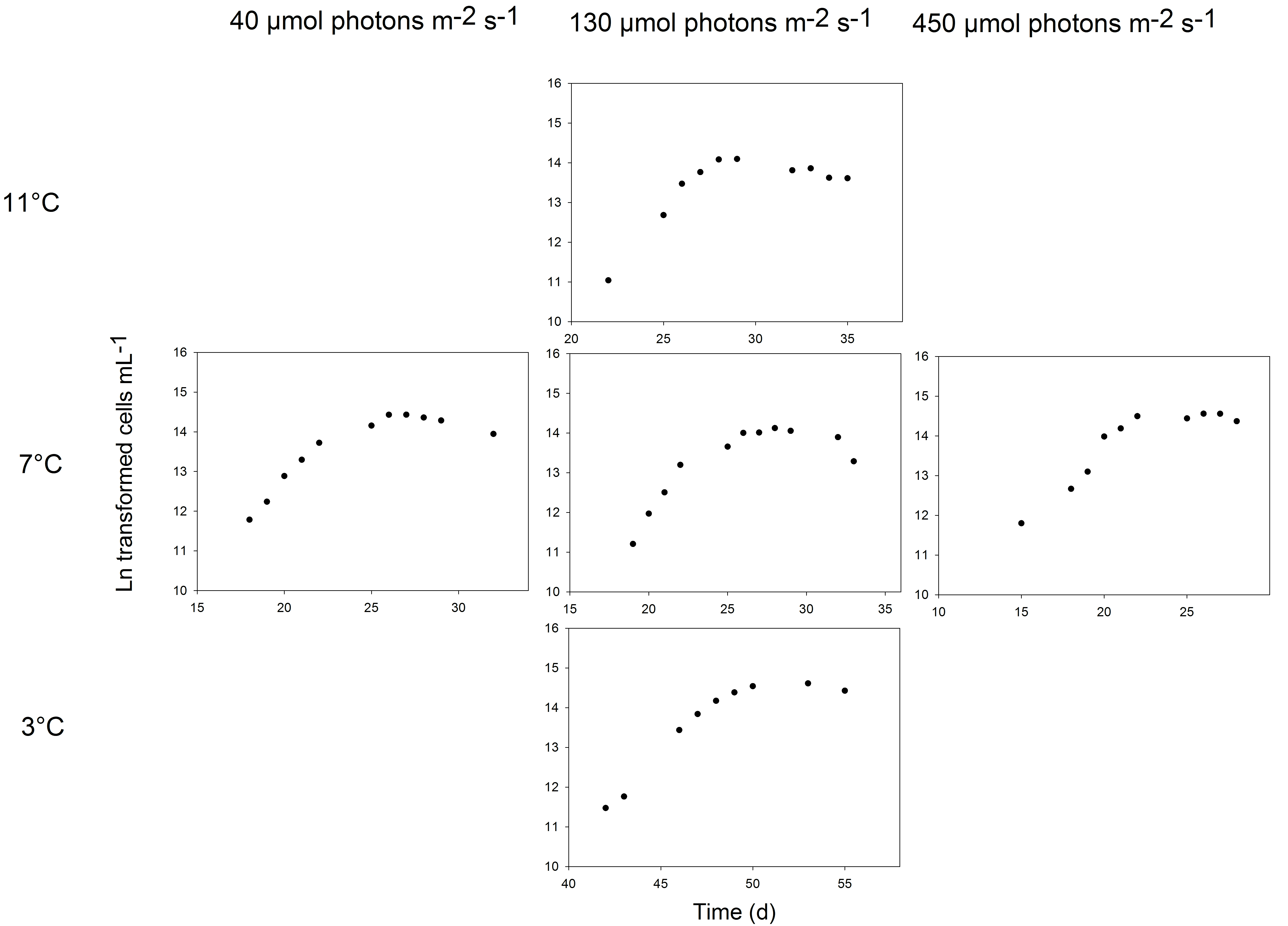

Supplement: S4 Fig — Increase in cell numbers during Si-limitation until the point of harvesting for the different combinations of light and temperature acclimation. The y-axis is the natural logarithm (ln) transformed cells mL-1. (TIF) [file pone.0126308.s004.tif]
